# Effect of Vitamin A on *Listeria monocytogenes* Infection in a Silkworm Model

**DOI:** 10.1371/journal.pone.0163747

**Published:** 2016-09-26

**Authors:** Yussaira Castillo, Jin Suzuki, Kenta Watanabe, Takashi Shimizu, Masahisa Watarai

**Affiliations:** The United Graduate School of Veterinary Science, Yamaguchi University, 1677-1 Yoshida, Yamaguchi 753-8515, Japan; Institute of Plant Physiology and Ecology Shanghai Institutes for Biological Sciences, CHINA

## Abstract

Insect infection models have been used increasingly to study various pathogenic agents in evaluations of pathogenicity and drug efficacy. In this study, we demonstrated that larvae of the silkworm *Bombyx mori* are useful for studying *Listeria monocytogenes* infections in insects. Infection with the *L*. *monocytogenes* wild-type strain induced silkworm death. Infection by a listeriolysin O (LLO) deletion mutant also induced silkworm death, but the bacterial numbers in silkworms were lower than those of the wild-type strain. Intracellular growth was observed when the silkworm ovary-derived cell line BmN4 was infected with the wild-type strain. Explosive replication was not observed in BmN4 cells infected with the LLO mutant and the bacterial numbers of the LLO mutant were lower than those of the wild-type strain. Pretreatment with vitamin A did not affect silkworm mortality after bacterial infection, but the efficiency of infecting the hemocytes and BmN4 cells was decreased with vitamin A treatment. Our results indicate that silkworm larvae are a useful insect infection model for *L*. *monocytogenes* and that vitamin A has protective effects against bacterial infection in silkworms.

## Introduction

Studying host-pathogen interactions and their related factors is of major importance for understanding the molecular mechanisms of human infectious diseases. Model systems are essential for investigating the complex processes of infectious diseases in humans. *Listeria monocytogenes* is a major food-borne pathogen that causes listeriosis, which is an invasive disease that can lead to meningitis, meningoencephalitis, septicemia, and abortion in its severest form [1]. Listeriosis occurs primarily in pregnant women, newborn infants, and the elderly as well as in immunocompromised patients, with a mortality rate of about 30%. The pathogen has a facultative intracellular life cycle, with the capacity for cellular invasion, intracellular replication and movement from cell to cell without an extracellular phase [2]. Rodents have been established as a useful model for analyzing the systemic phase of *L*. *monocytogenes* infections [3], but costs and ethical issues are associated with the use of mammalian hosts, and thus non-mammalian models have proved useful surrogate hosts [4]. Some non-mammalian studies have contributed to our understanding of the infectious process of *Listeria* species. The nematode *Caenorhabditis elegans* was the first invertebrate model to be employed [5], followed by the larvae and adults of the fruit fly *Drosophila melanogaster* [6,7], and the larvae of the wax moth (*Galleria mellonella*), which has been validated as an alternative host model for analyzing *L*. *monocytogenes* [8,9]. The larvae of *G*. *mellonella* have a significant advantage compared with previous models because they can be incubated at human body temperature (37°C).

In recent years, the larvae of *Bombyx mori*, commonly known as the silkworm, have been employed as an infection model for a large variety of agents including bacteria, viruses, and fungi [10–13]. The silkworm has been utilized to produce silk for more than 4,000 years, so there is a large volume of accumulated technical information about this insect. Therefore, the silkworm has various advantages compared with other insects, such as its easy handling, low cost, and ready availability.

Vitamins are one of the various organic compounds used by organisms and limited amounts of them are essential for natural performance. Vitamins are essential for silkworm and its direct supplementation to the insect body have been reported to affect the larvae metabolism [14].

The pathogenesis of *L*. *monocytogenes* has been studied using insects, but a comparative analysis of the pathogenic potential of a listeriolysin O (LLO) mutant has not been addressed previously in silkworms. Thus, in the present study, we explored the possibility of using silkworms as an infection model for *L*. *monocytogenes*. We examined whether the mutants could kill silkworms in an effective manner at room temperature. In addition, we examined whether vitamin A, an essential nutritional requirement with major effects on the immune system by extending both innate and adaptive immune responses [14], can offer protection to silkworms during the infection process.

## Materials and Methods

### Bacterial strains and culture conditions

*Listeria monocytogenes* strain EGD, an LLO deletion mutant (*Δhly*), and an LLO-complemented strain (*Δhly*::*hly*) were used in this study [15]. The *L*. *monocytogenes* strains were maintained in frozen glycerol stocks and cultured overnight in brain-heart infusion (BHI) broth (Becton Dickinson, Franklin Lakes, NJ, USA) at 37°C with shaking, or on BHI broth containing 1.5% agar.

### Vitamin A supplementation in silkworms

Fourth instar *Bombyx mori* larvae (Hu·Yo × Tukuba·Ne) were obtained from Ehime-Sanshu (Ehime, Japan). The larvae were raised by feeding them with Silkmate (Nosan Corporation, Kanagawa, Japan) at room temperature. Fifth instar larvae weighing 3.2–3.8 g were injected into the dorsolateral hemocoel with a single dose of 0.1 mL vitamin A acetate (MP Biomedicals, LLC) at 2.25 mg/mL (1,250 IU/mL) dissolved in phosphate-buffered saline (PBS). Pressure was applied immediately at the injection site by using a finger for 10 s to stop the bleeding. Syringes (1 mL) and needles (27G × 3/4″) were obtained from Terumo Inc., Tokyo, Japan.

### Infection of silkworms with *L*. *monocytogenes*

Bacterial inocula were injected dorsolaterally into the hemocoel of pretreated and control (3.2–3.8 g body weight) last-instar larvae using 1 mL disposable syringes. Fifth instar larvae were injected with 0.05 mL of a bacterial suspension containing approximately 10^4^ cells of *L*. *monocytogenes* EGD, the LLO deletion mutant, or the LLO-complemented strain in PBS. After injection, the larvae were incubated with food at 25°C. Caterpillars were considered dead when they exhibited no movement in response to touch. The day of infection was considered to be day 0 of infection. To obtain colony-forming unit (CFU) counts at 1 h, 24 h, and 48 h post infection, the infected silkworm larvae were weighed and placed in a disposable 15 mL centrifuge tube, chopped up with scissors, homogenized with a disposable homogenizer (Biomasher SP (EOG-sterellied), Funakoshi Co. Ltd, Tokyo, Japan) and suspended in 3 mL of PBS. Subsequently, the suspension was centrifuged at 300 × *g* for 30 s and solid tissues were separated from the concentrated suspension. After appropriate dilutions, the sample suspensions were spread on BHI agar plates, incubated overnight, and the numbers of colonies were counted. To calculate the CFU count, the summed volumes of hemolymph and tissues were estimated together (1 g = 1 mL). The number of hemocytes was counted in six independent fields of view by confocal laser scanning microscopy, and the relative hemocyte number was calculated as the ratio of each group relative to that of the control. The larvae were maintained at room temperature with food post infection. All of the experiments were replicated at least three times independently and examples of representative results are reported.

### Cell culture

The *Bombyx mori* ovary-derived cultured cell line BmN4 was a gift from Dr. Kusakabe [16]. BmN4 cells were cultured in IPL-41 insect medium (Sigma-Aldrich, St. Louis, MO) supplemented with 10% fetal bovine serum (FBS) at 25°C in the atmosphere. BmN4 cells were seeded (1 × 10^5^ per well) in 48-well tissue culture plates coated with concanavalin A at 48 h prior to use. Plate coating was performed according to the following steps: the bottoms of the wells were covered with 100 mM concanavalin A and incubated for 2 h at room temperature, before removing the concanavalin A solution, air drying completely, and washing once with PBS.

### Efficiency of bacterial invasion and intracellular replication

BmN4 cells were grown in 24-well tissue culture plates for 24 h to reach confluence. For the supplemented treatment, IPL-41 medium containing vitamin A (0.45, 2.25, or 11.25 mg/ml) was added to the BmN4 cells at 24 h before infection. Bacterial strains were deposited onto BmN4 cells at a multiplicity of infection (MOI) of 10 by centrifugation at 150 × *g* for 10 min at room temperature. To measure the invasion rate, after incubating for 2 h at 25°C, the cells were washed twice with PBS, and then incubated in PBS containing gentamicin (100 μg/mL, Sigma) for 30 min. The cells were then washed two times with PBS and incubated for 30 min in IPL-41 medium containing vitamin A (when supplementation was performed) and gentamicin (25 μg/mL). Next, the cells were washed three times with PBS and lysed with cold distilled water. The CFU counts were determined on BHI plates by serial dilution. To measure the intracellular replication efficiency, the infected cells were incubated at 25°C for 2 h, washed twice, and incubated again in PBS containing gentamicin (100 μg/mL) for 30 min. The cells were then washed and incubated for 24 h in IPL-41 medium containing vitamin A and gentamicin (25 μg/mL). The cell washing, lysis, and plating procedures were the same as those used in the bacterial invasion efficiency assay.

### Immunoblotting

Each strain of *L*. *monocytogenes* was cultured in BHI broth supplemented with PBS and 0.45, 2.25, or 11.25 mg/ml of vitamin A acetate for 8 h or 12 h at 25°C or 37°C. Each supernatant was collected after centrifugation (16,000 × *g*, 4°C) for 30 min. After separating each protein from 5 μL of culture supernatant by SDS-PAGE with 10% polyacrylamide gel, the proteins were transferred onto polyvinylidene difluoride membranes (Millipore, Billerica, MA). After blocking with 5% nonfat dry milk Tris-buffered saline (TBS) at room temperature for 2 h, the membranes were incubated overnight with anti-LLO antibody (1:1,000; ab43018; Abcam plc, Cambridge, UK) at 4°C. After washing with TBS containing 0.02% (v/v) Tween 20, the membranes were incubated for 1 h with horseradish peroxidase-conjugated secondary antibody (0.01 μg/mL) at room temperature and immunoreactions were visualized using the enhanced chemiluminescence detection system (GE Healthcare Life Science, Little Chalfont, UK).

### Fluorescence microscopy

Aliquots of hemolymph (200 μL) were harvested from supplemented and non-supplemented infected larvae under sterile conditions and deposited onto a 24-well tissue culture plate on sterile coverslips, which contained 500 μL IPL-41 insect medium supplemented with 10% FBS, and were incubated at 25°C in the atmosphere. Hemocytes were promptly centrifuged at 150 × *g* for 5 min, washed twice with PBS and incubated at room temperature with FBS-supplemented IPL-41 medium for 15 min. The hemocytes were then washed twice with PBS, fixed with 4% paraformaldehyde in PBS for 30 min at room temperature, and washed twice with PBS. The samples were permeabilized with 0.2% Triton X-100, washed twice with PBS, and then treated with 1 μM rhodamine phalloidin and 5 μg/mL anti-*L*. *monocytogenes* rabbit antibody (Viro Stat, Portland, ME) diluted in blocking buffer (5% bovine serum albumin in PBS) to identify intracellular bacteria. Fluorescent images were obtained using a FluoView FV100 confocal laser scanning microscope (Olympus Tokyo, Japan).

### Statistical analysis

Statistical analyses were performed using the Student’s t-test or one-way ANOVA with the post hoc Tukey–Kramer test. Statistically significant differences between groups were accepted at *P* < 0.05 in three identical experiments. The survival curves were estimated with the Kaplan-Meier method and the log-rank test was used to determine significant differences (*P* < 0.05).

## Results

### Infection of silkworm larvae with human pathogenic *L*. *monocytogenes*

**Fig 1:**
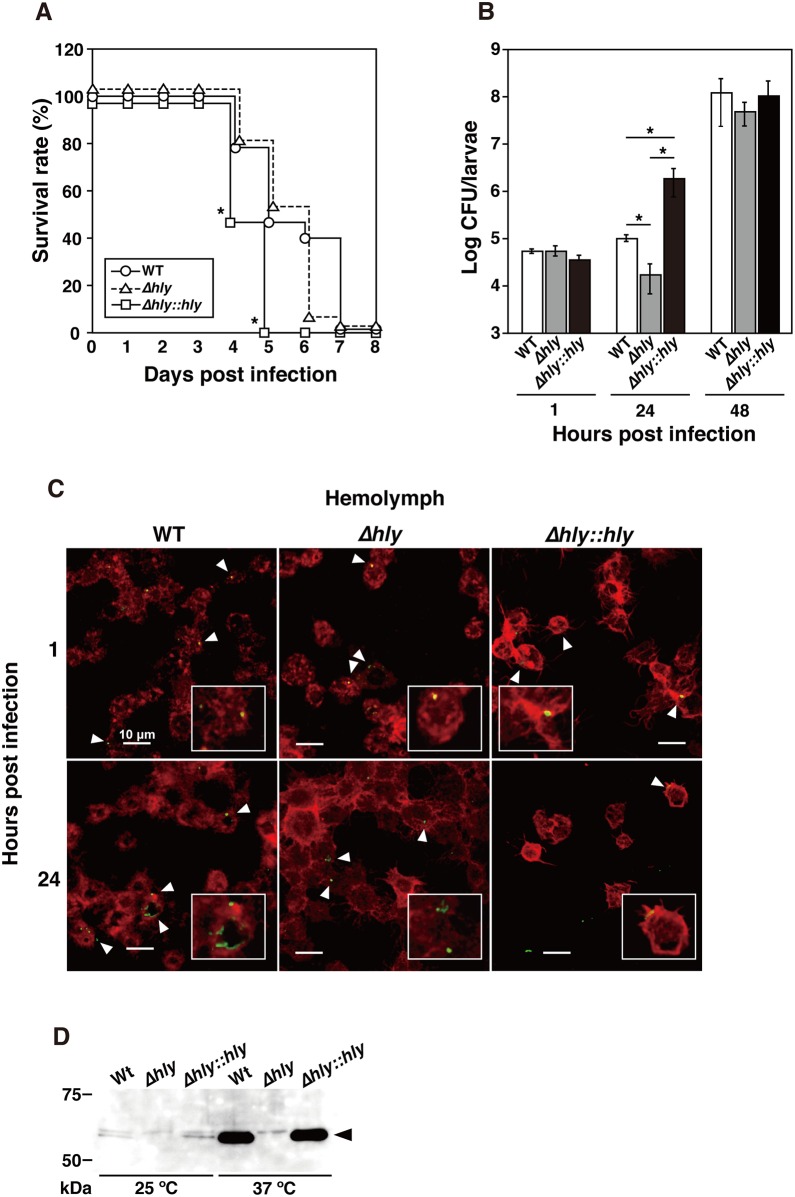
Infection of silkworms with *L*. *monocytogenes*. **(A)** Survival rate of silkworms. Fifteen insects per group were infected and monitored to determine the time required to kill 50% of the silkworms. Approximately 10^4^ bacteria belonging to the wild-type strain (WT, circles), LLO deletion mutant (*Δhly*, triangles), and LLO-complemented strain (*Δhly*::*hly*, squares) were sufficient to kill 50% (LD50) of the silkworms within 4 and 5 days. Significant differences were accepted at *P* < 0.05 and they are indicated by asterisks (*). **(B)** The bacterial numbers of each strain detected in silkworms at 48 h post inoculation. The data represent averages based on triplicate samples from three identical experiments and the error bars represent the standard error of the mean (SEM) (n = 9). Significant differences were accepted at *P* < 0.05 and they are indicated by asterisks (*). **(C)** Hemocytes (red) containing *L*. *monocytogenes* strains (green, arrowheads) were observed by confocal laser scanning microscopy at 1 h and 24 h post inoculation. Scale bar represents 10 μm. **(D)** Immunoblot analysis of LLO expression. Samples were prepared from the wild-type strain, LLO deletion mutant, and LLO-complemented strain cultivated at 37°C or 25°C in BHI broth.

In order to determine whether the silkworm *B*. *mori* can be used as a model system for *L*. *monocytogenes* infection, we first examined the infectivity of wild-type *L*. *monocytogenes* EGD, an LLO deletion mutant, and an LLO-complemented strain at room temperature. *L*. *monocytogenes* was serially diluted and then injected into 15 individual silkworms per group, before monitoring the time required to kill 50% of the silkworms (LT50). The PBS-injected larvae survived for at least six days and subsequently matured into pupae (data not shown). The LT50 was determined when approximately 10^4^ cells of the wild-type strain, LLO deletion mutant, or LLO-complemented strain were injected at room temperature. The wild-type strain and LLO-complemented strain obtained LT50 values of 120 h and 96 h (five and four days), respectively (Fig 1A). A delay in the LT50 (approximately six days) was observed for the LLO deletion mutant. All of the infected larvae looked normal for at least two days and the activity then decreased, before they stopped eating and finally died. Their skin color became dark only after death.

### Growth of *L*. *monocytogenes* in silkworms

To determine whether *L*. *monocytogenes* could proliferate in infected larvae, we analyzed the hemolymph and tissues isolated from the larvae injected with the wild-type strain, LLO deletion mutant, or LLO-complemented strain. To determine the CFU counts per larva, bacteria were harvested from infected fifth instar larvae and the bacterial colony counts were quantified. The CFU counts showed that the bacterial numbers of the three strains were at the same level 1 h after the initial inoculation (Fig 1B). However, at 24 h post injection, there was a rapid increase in the bacterial number of the LLO-complemented strain compared with the wild-type strain. The proliferation of the *L*. *monocytogenes* LLO mutant decreased following the initial inoculation but recovered to the same levels as the wild-type strain and the LLO-complemented strain at 48 h after inoculation (Fig 1B).

To further understand the host-pathogen interactions in this silkworm model, we examined hemocyte isolated from infected larvae using confocal microscopy. Intracellular bacteria belonging to the three strains were observed at 1 h and 24 h after infection (Fig 1C). Infection with the LLO-complemented strain decreased the number of hemocytes by 55.3% ± 9.7% compared with those injected with the wild-type strain and LLO deletion mutant at 24 h after infection. These results demonstrate that the LLO produced by *L*. *monocytogenes* contributes to bacterial proliferation in the silkworm. We also confirmed the *in vitro* expression of LLO at room temperature by immunoblotting. High levels of LLO expression were observed with the wild-type strain and LLO-complemented strain at 37°C and slightly lower LLO expression at 25°C (Fig 1D). LLO expression was not detected in the LLO deletion mutant at 37°C and 25°C.

### Intracellular replication of *L*. *monocytogenes* in BmN4 cells

**Fig 2:**
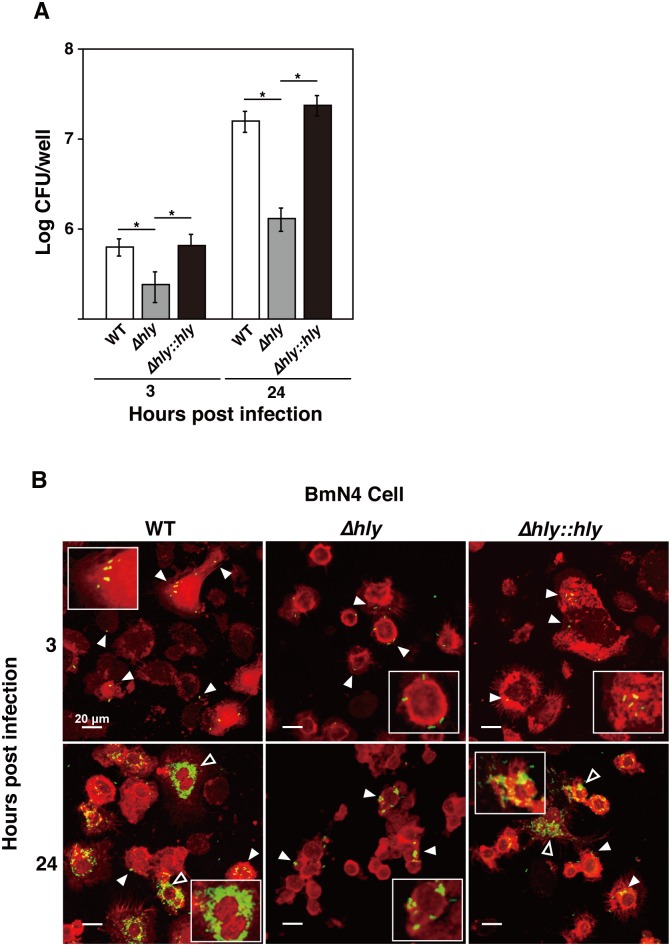
Intracellular replication of *L*. *monocytogenes* in BmN4 cells. **(A)** *L*. *monocytogenes* replicates intracellularly in BmN4 cells. The silkworm ovary-derived cell line BmN4 was infected with the wild-type strain (WT), LLO deletion mutant (*Δhly*), and LLO-complemented strain (*Δhly*::*hly*) at an MOI of 10. Bacterial invasion and intracellular replication by each strain were measured at 3 h and 24 h after infection. The data represent averages based on triplicate samples from three identical experiments and the error bars represent the standard deviations. Significant differences were accepted at *P* < 0.05 and they are indicated by asterisks (*). **(B)** BmN4 cells (red) containing *L*. *monocytogenes* strains (green, white arrowheads) were observed by confocal laser scanning microscopy at 3 h and 24 h post inoculation. Scale bar represents 20 μm. Explosive bacterial replication is indicated by open arrowheads. The experiment was replicated three times independently.

It well known that *L*. *monocytogenes* is an intracellular pathogen. To examine the intracellular replication kinetics of *L*. *monocytogenes*, we infected the silkworm ovary-derived cell line BmN4 with the wild-type strain, LLO deletion mutant, and LLO-complemented strain at an MOI of 10. We measured the bacterial invasion and intracellular replication of each strain at 3 h and 24 h after infection. The bacterial count of the LLO deletion mutant was significantly lower in BmN4 cells compared with those of the wild-type strain and LLO-complemented strain at 3 h and 24 h post infection (Fig 2A). Intracellular replication by all three strains was observed at 24 h post infection.

Next, we analyzed intracellular replication by the bacteria using confocal microscopy, where we observed the wild-type strain, LLO deletion mutant, and LLO-complemented strain in BmN4 cells at 3 h post infection (Fig 2B). Explosive replication was observed in BmN4 cells infected with the wild-type strain and LLO-complemented strain, but low level replication was detected in BmN4 cells infected with the LLO deletion mutant at 24 h post infection (Fig 2B).

### Effects of vitamin A pretreatment on *L*. *monocytogenes* infections in silkworms

**Fig 3:**
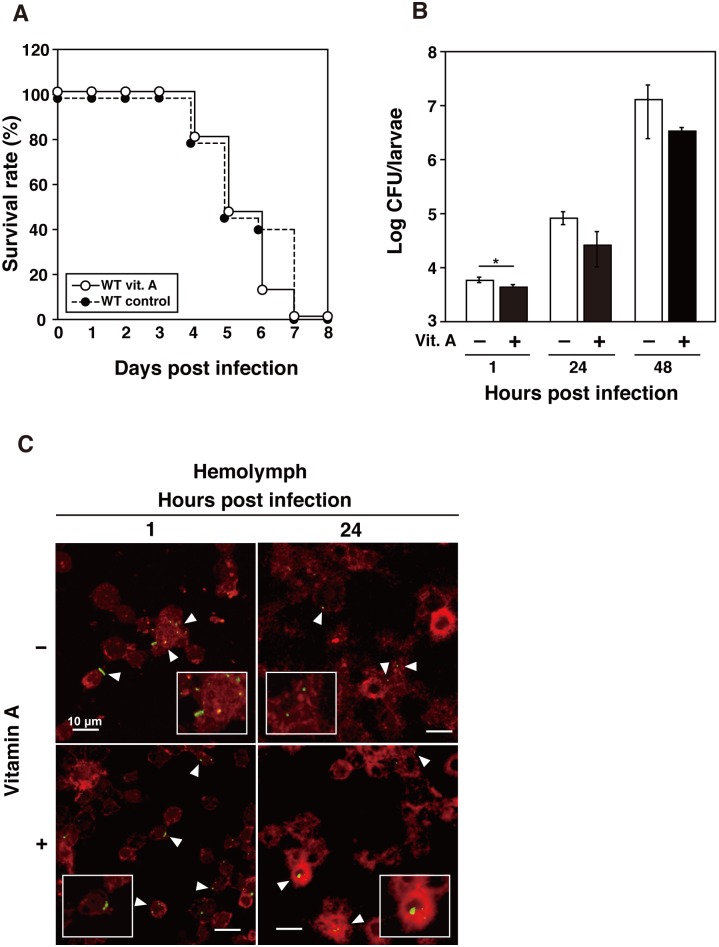
Infection of *L*. *monocytogenes* to silkworm pretreated with vitamin A. **(A)** Survival of infected silkworms after previous supplementation with vitamin A. Approximately 10^4^ bacteria of the wild-type strain were used to infect silkworms pretreated with (open circles) or without vitamin A (black circles). Groups of five insects per group were checked daily for survival. Survival was observed until 8 days post infection. **(B)** Bacterial numbers of the wild-type strain in silkworms pretreated with (black bars) or without vitamin A (open bars) until 48 h post inoculation. The data represent averages based on triplicate samples from three identical experiments and the error bars represent the SEM (n = 9). Significant differences were accepted at *P* < 0.05 and they are indicated by asterisks (*). **(C)** Hemocytes (red) containing *L*. *monocytogenes* strains (green, arrowheads) isolated from silkworm pretreated with or without vitamin A were observed by confocal laser scanning microscopy at 3 h and 24 h post inoculation. Scale bar represents 10 μm.

The innate immune system of the silkworm is considered to function at both the cellular and humoral levels. The role of vitamin A in the immune system extends to both innate and adaptive immune responses in vertebrates [14]. At first, we check the effects of vitamin A treatment in non-infected silkworms. No significant differences were observed in the health conditions, such as body weight, feeding activity and time to pupation, between the silkworms pretreated with vitamin A and control (data not shown). To examine the effects of vitamin A supplementation on *L*. *monocytogenes* infections in silkworms, we infected vitamin A-pretreated larvae with approximately 10^4^ cells of the wild-type strain at 12 h after supplementation, and we then measured the survival rates of the supplemented silkworms and their CFU counts. There was no significant difference between the vitamin A-supplemented silkworms and the control (Fig 3A). There was a one-day delay in mortality between the two groups. Next, we determined the bacterial numbers in infected silkworms based on the CFU counts. To measure the bacterial numbers in infected silkworms, the larvae were infected with the wild-type strain and the bacterial counts were determined at 1 h, 24 h, and 48 h after infection. The number of bacteria was significantly lower in vitamin A-supplemented silkworms compared with the non-supplemented silkworms (Fig 3B). This effect was maintained throughout the course of infection. To investigate the effect of vitamin A supplementation on bacterial invasion into the hemocyte of silkworms, we examined hemocyte isolated from infected larvae using confocal microscopy. Intracellular bacteria were observed at 1 h and 24 h after infection, and there were no differences between vitamin A-supplemented and non-supplemented silkworms (Fig 3C).

### Effects of vitamin A pretreatment on *L*. *monocytogenes* infection in BmN4 cells

**Fig 4:**
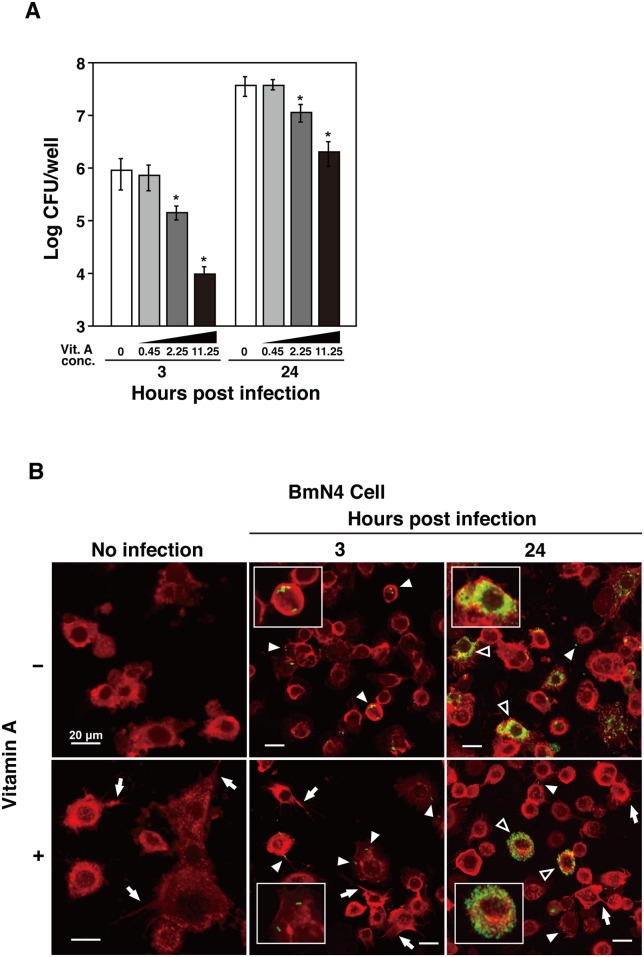
*L*. *monocytogenes* infection of BmN4 cells after pretreatment with vitamin A. **(A)** Pretreatment with vitamin A inhibited *L*. *monocytogenes* infection in BmN4 cells. BmN4 was infected with the wild-type strain at a multiplicity of infection of 10. Bacterial invasion and intracellular replication were measured at 3 h and 24 h after infection in BmN4 cells at the indicated vitamin A concentrations (mg/ml). The data represent averages based on triplicate samples from three identical experiments and the error bars represent the standard deviations. Significant differences were accepted at *P* < 0.05 and they are indicated by asterisks (*). **(B)** BmN4 cells (red) containing *L*. *monocytogenes* strains (green, white arrowheads) were observed by confocal laser scanning microscopy at 3 h and 24 h post inoculation. BmN4 cells were pretreated with (11.25 mg/ml) or without vitamin A. Scale bar represents 20 μm. Explosive bacterial replication is indicated by open arrowheads. Filopodia-like structure are indicated by arrows. The experiment was replicated three times independently.

To determine the effects of vitamin A on non-hemocyte silkworm cells, we infected BmN4 cells with the wild-type strain at an MOI of 10 and we measured bacterial invasion and intracellular replication at 3 h and 24 h after infection. We found that the number of intracellular bacteria decreased at 3 h and 24 h after infection in a dose-dependent manner (Fig 4A). Pretreatment with vitamin A significantly reduced bacterial invasion at concentrations of 2.25 mg/ml and 11.25 mg/ml.

Next, we analyzed intracellular replication by the bacteria using confocal microscopy. The wild-type strain was observed in BmN4 cells treated with or without vitamin A at 3 h post infection, but the number of intracellular bacteria was lower in BmN4 cells treated with vitamin A (Fig 4B, arrowheads). Explosive replication was observed in BmN4 cells treated with or without vitamin A at 24 h post infection (Fig 4B, open arrowheads), thereby suggesting that vitamin A treatment did not inhibit intracellular replication by the bacteria.

Vitamin A is known for its cytotoxic effects on vertebrate cells [17], but there were no effects on the viability of BmN4 cells at the vitamin A concentration used in this study (11.25 mg/ml). Confocal microscopy demonstrated that the shape of the BmN4 cells was changed by vitamin A treatment and filopodia-like structures were observed in BmN4 cells treated with vitamin A (Fig 4B arrows).

## Discussion

Recently, insect infection models have been used to study various human infectious diseases including *L*. *monocytogenes* [9]. However, insect models have been criticized for being too “distant” from human disease processes. The strongest aspects of this argument are: (i) the temperature ranges employed, which means that certain pathogens cannot express virulence factors; and (ii) physiological problems, such as the absence of the same target organs [18]. Bacterial infections can cause opportunistic diseases and innate immunity has a major role in combating these diseases. Silkworms may provide a useful model for analyzing innate immunity because insects and mammals share common innate immune mechanisms. Thus, silkworms provide a model for studying host-pathogen interactions and cellular infection in the presence of innate immunity. Non-mammalian models have logistical, budgetary, and ethical advantages over mammals. In particular, the ease and low costs required to obtain large numbers of larvae facilitate large-scale screening, which would be prohibitive in mammals. In addition, the amounts of drugs needed to examine therapeutic effects are generally much smaller in invertebrates than in mammals due to their low body weight. Other significant benefits of this model are that *B*. *mori* can be incubated at room temperature (25°C) and the specific virulence factors of *L*. *monocytogenes* that are active at this temperature can be found. In the present study, we found that silkworm larvae were killed by infections with the *L*. *monocytogenes* wild-type strain, LLO deletion mutant, and LLO-complemented strain when infected at room temperature. LLO is a major virulence factor, which was originally shown to be crucial for bacterial escape from the internalization vacuole after entry into the host cell [2]. The delay until bacterial proliferation in silkworms infected with the LLO deletion mutant and the number of hemocytes were reduced dramatically when silkworms were infected with the LLO-complemented strain, which indicates that LLO may contribute to the early stage of infection in silkworms. Proliferation of the LLO deletion mutant and the wild-type strain in silkworms as well as the slight expression of LLO were shown in an *in vitro* experiment at 25°C, and thus LLO may have a limited role in bacterial infection so mortality may require bacterial growth in silkworms.

Recently, an insect infection model using *Galleria mellonella* was employed to study *L*. *monocytogenes* pathogenesis [8,9], which showed that the production of LLO is necessary for toxicity and bacterial growth [9]. The virulence of strains with mutated LLO production was attenuated completely and these strains did not cause mortality in the insect. The results obtained using our silkworm infection model differed from those with the previous insect infection model because we maintained the silkworms at 25°C. Silkworms appeared unhealthy when maintained at 37°C so it would have been difficult to perform experiments at 37°C under our test conditions. Therefore, this model can be useful for investigating virulence factors that are not regulated by temperature. Silkworms may also be suitable for analyzing environmental infection mechanisms. It is expected that novel virulence factors involved in insect infections will be identified using the silkworm model.

A visible phenotype associated with bacterial infection in insects is increased pigmentation in the larvae bodies, which usually indicates pro-phenoloxidase (PPO) induction to cause melanization [19,20]. In silkworms, melanization has been documented in infections by both Gram-negative (*Pseudomonas aeruginosa*, *Escherichia coli*, and *Vibrio cholerae*) and Gram-positive bacteria (*Staphylococcus aureus*) [13]. In this silkworm model, *L*. *monocytogenes* killed silkworms without inducing melanization (data not shown). This may suggested that *L*. *monocytogenes* escape from immune responses of silkworm and proliferate rapidly. However, further study is needed to demonstrate if *L*. *monocytogenes* posses a mechanism for inhibiting the induction of melanization.

In recent years, the *B*. *mori* ovary-derived BmN4 cell line has been used to study viral replication and the development of antiviral compounds [21,22]. To the best of our knowledge, this is the first study to use the BmN4 cell line for investigating intracellular replication by *L*. *monocytogenes in vitro*. We observed intracellular spreading in BmN4 cells infected with the wild-type strain and LLO-complemented strain, but not with the LLO deletion mutant. These results suggest that LLO has an important role in escaping from vacuoles in BmN4 cells. The intracellular replication phenotype differs between hemocytes and BmN4 cells. LLO may have strong cytotoxic effects on hemocytes, so intracellular spreading was not observed clearly. Intracellular replication in other types of cells and the extracellular replication of *L*. *monocytogenes* may be direct factors that lead to the death of silkworms rather than the intracellular replication of the bacterium.

Vitamins are essential dietary components and they influence the immune system [23]. Especially, vitamin A shows immunomodulatory function [24] and enhance resistance to various infectious diseases [25]. For example, vitamin A plays an important role in decrease of *Toxoplasma gondii* or *Mycobacterium tuberculosis* [26,27]. In addition, we previously demonstrated that *all-trans* retinoic acid, the active form of vitamin A, induces protection of the host against *L*. *monocytogenes* infection in a murine model and that vitamin A may have a bacteriostatic effect on *L*. *monocytogenes* [28]. Therefore, we hypothesized that vitamin A supplementation in silkworms might enhance the host immune system. Multi-vitamin supplementation with vitamins C, B12, D3, and A has been used in the silkworm industry to enhance biological and economical parameters (larval weight, female cocoon and pupal weight, and egg productivity) with good results [29]. Due to the cytotoxic properties of vitamin A, our main concern when using insects as an infection model was a possible difference in drug toxicity and metabolism between invertebrates and mammals. However, some studies have reported that the LD50 values for cytotoxic substances in silkworms are consistent with those in mammals [30]. Our results showed that vitamin A only had limited protective effects during the early stage of infection in silkworms. Insects do not possess an acquired immune system where antibodies have a significant role [20], which might influence the chemotherapeutic effects of vitamin A. In mammals, vitamin A affects adaptive immune-cell subsets and general lymphocyte functions. In this study, we observed that vitamin A treatment changed the shape of BmN4 cells, thereby suggesting that vitamin A may induce cell activation and differentiation in BmN4 cells. However, our understanding of the metabolite conjugation pathways is quite limited in silkworms and further investigations are needed.

In this study, we developed silkworm infection model with *L*. *monocytogenes*. Using this model, we demonstrated that *L*. *monocytogenes* is able to replicate intracellulary, and LLO is an important pathogenic factor in invertebrates. Moreover, vitamin A is an inhibitory agents against *L*. *monocytogenes* infection. Although, it is unclear invertebrates play an important role for survive of *L*. *monocytogenes* in environment, the results of this study may help to prevent the spread of *L*. *monocytogenes* in the environment.
